# Novel insights into P450 BM3 interactions with FDA-approved antifungal azole drugs

**DOI:** 10.1038/s41598-018-37330-y

**Published:** 2019-02-07

**Authors:** Laura N. Jeffreys, Harshwardhan Poddar, Marina Golovanova, Colin W. Levy, Hazel M. Girvan, Kirsty J. McLean, Michael W. Voice, David Leys, Andrew W. Munro

**Affiliations:** 1 Centre for Synthetic Biology of Fine and Specialty Chemicals (SYNBIOCHEM), Manchester Institute of Biotechnology, School of Chemistry, The University of Manchester, Manchester, M1 7DN United Kingdom; 2 Manchester Protein Structure Facility (MPSF), Manchester Institute of Biotechnology, School of Chemistry, The University of Manchester, Manchester, M1 7DN United Kingdom; 3 Cypex Ltd., 6 Tom McDonald Avenue, Dundee DD2 1NH, Scotland, United Kingdom

## Abstract

Flavocytochrome P450 BM3 is a natural fusion protein constructed of cytochrome P450 and NADPH-cytochrome P450 reductase domains. P450 BM3 binds and oxidizes several mid- to long-chain fatty acids, typically hydroxylating these lipids at the ω-1, ω-2 and ω-3 positions. However, protein engineering has led to variants of this enzyme that are able to bind and oxidize diverse compounds, including steroids, terpenes and various human drugs. The wild-type P450 BM3 enzyme binds inefficiently to many azole antifungal drugs. However, we show that the BM3 A82F/F87V double mutant (DM) variant binds substantially tighter to numerous azole drugs than does the wild-type BM3, and that their binding occurs with more extensive heme spectral shifts indicative of complete binding of several azoles to the BM3 DM heme iron. We report here the first crystal structures of P450 BM3 bound to azole antifungal drugs – with the BM3 DM heme domain bound to the imidazole drugs clotrimazole and tioconazole, and to the triazole drugs fluconazole and voriconazole. This is the first report of any protein structure bound to the azole drug tioconazole, as well as the first example of voriconazole heme iron ligation through a pyrimidine nitrogen from its 5-fluoropyrimidine ring.

## Introduction

The cytochromes P450 (P450s or CYPs) are a superfamily of heme *b*-binding enzymes that catalyze the oxidative modification of a huge number of organic substrates^1^. This is typically achieved through the formation of a highly reactive heme iron-oxo species known as compound I (a ferryl-oxo [Fe^IV^ = O] porphyrin radical cation species) that inserts an oxygen atom into a substrate bound close to the heme in the P450 active site^2,3^. According to the nature of the substrate involved and its binding mode in the active site, various different catalytic outcomes can occur following the oxidation of the substrate, including hydroxylation, demethylation/dealkylation, epoxidation, deamination, decarboxylation and sulfoxidation^4,5^.

Human P450s play crucial roles including the metabolism and interconversion of steroids, the hydroxylation/epoxidation of saturated and unsaturated fatty acids (including prostaglandins), and the oxidation of pharmaceuticals and other xenobiotics to facilitate their metabolism and excretion^6–8^. The steroid oxidizing human P450s typically exhibit strict substrate selectivity and regioselectivity in their reactions, whereas many of the xenobiotic transforming P450s are known to be more promiscuous in substrate binding and can catalyze diverse types of oxidative reactions. Perhaps the best example of the latter class of human P450s is CYP3A4, which is an extremely versatile enzyme that is able to metabolize a large number of drug molecules, including tamoxifen, erythromycin, codeine, steroids and a number of statin drugs^9^. The conformational flexibility of CYP3A4 and its adaptability to accommodate and oxidize substrates of different sizes and chemical properties makes this P450 a prodigious catalyst in this enzyme superfamily.

In humans, the P450s are located in the endoplasmic reticulum of cells, or in the mitochondrial inner membrane of adrenal glands (and other steroidogenic tissues), liver and kidneys^9^. Human and other eukaryotic P450s are membrane-associated enzymes attached to membranes through an N-terminal transmembrane alpha-helical segment. These P450s present some challenges in protein purification in view of the need for detergents to solubilize the enzymes, and due to limited yields of these P450s. In contrast, P450s from bacteria and archaea are soluble enzymes that are devoid of membrane “anchor” regions^10^. These enzymes are usually soluble and located in the cell cytoplasm, and they can typically be expressed in much higher amounts than their eukaryotic counterparts^11,12^. Among the bacterial P450s, one of the best characterized enzymes is the *Bacillus megaterium* CYP102A1 (P450 BM3), which Armand Fulco’s group identified as a fatty acid hydroxylase that could catalyze the hydroxylation of saturated fatty acid substrates, primarily at the ω-1, ω-2, and ω-3 positions^13^. P450 BM3 (BM3) is a natural fusion of a cytochrome P450 (N-terminal) to a FAD-, FMN- and NADP(H)-binding cytochrome P450 reductase (CPR). The BM3 CPR resembles the membrane-associated eukaryotic CPRs that transfer electrons to their cognate P450 enzymes, but is a soluble protein devoid of a membrane anchor region. BM3 has the highest catalytic rate for substrate oxidation yet reported for a P450 monooxygenase at ~285 s^−1^ with arachidonic acid as the substrate^14^. The component P450 and CPR domains of BM3 were successfully expressed in isolation, although they no longer interacted efficiently for fatty acid hydroxylation^15,16^. In addition, the FAD/NADPH-binding (ferredoxin reductase-like) and FMN-binding (flavodoxin-like) modules were also produced in large amounts using *E*. *coli* expression systems^17^. Intact BM3 was shown to be a dimeric enzyme with NADPH-dependent electron transfer able to occur between the CPR domain of one monomer and the heme domain of the other in the BM3 dimer^18^.

Early studies on P450 BM3 demonstrated its high catalytic rate and selectivity towards medium- to long-chain fatty acid substrates. However, the catalytic proficiency of BM3 and its convenience as a self-sufficient catalyst (requiring only NADPH and substrate for activity) led various researchers to use protein engineering strategies in order to alter its substrate specificity. There have been a number of successful studies in this area in recent years, including the production of BM3 variants that can bind and hydroxylate propane to propanol, or that catalyze selective carbene transfer from diazoesters to olefins in intact *E*. *coli* cells^19,20^. Other researchers have developed mutants that can transform the sesquiterpene (+)-valencene into nootkatone and nootkatol products, with nootkatone being an important fragrance compound^21^. More recent work in our group has used the double mutant (DM) form of the flavocytochrome P450 BM3 enzyme (F87V/A82F), in which the first mutation expands available substrate binding space in the active site, while the second mutation is more distant from the heme but causes a structural readjustment in the P450 that alters its conformational state. The DM variant appears much more flexible than wild-type (WT) BM3, and can bind and oxidize drug molecules including omeprazole and related gastric proton pump inhibitors (PPIs) to produce human metabolites (e.g. 5-OH esomeprazole, rabeprazole desmethyl ether and lansoprazole sulfone) of these drugs^22,23^.

In view of the more promiscuous nature of the BM3 DM enzyme and its ability to bind a number of molecules that do not interact productively with WT BM3, we have explored the binding of a range of bulky azole antifungal drugs to the heme domain of the BM3 DM enzyme. These azole compounds typically have modest binding affinities for WT BM3, as evidenced by their inability to induce substantial heme spectral shifts that are indicative of either substrate-like or inhibitor-like P450 binding behavior. The azoles were developed as inhibitors of the fungal 14α-sterol demethylase (CYP51 family) enzymes, and characteristically enter the CYP51 active site and inhibit sterol demethylation by ligating to the P450 heme iron through a nitrogen atom from an imidazole or triazole group on the drug. An indirect heme iron binding mode, in which an azole nitrogen makes hydrogen bonding interactions with a 6^th^ ligand water molecule retained on the heme iron, has also been reported in a small number of cases^24,25^. Over time many pathogenic fungi have developed resistance to various drugs from the azole class (e.g. *Aspergillus fumigatus*) and multidrug resistance to azoles and many other antifungal compounds (e.g. in *Candida auris* and *Candida glabrata*)^26^. Many new azoles are produced and marketed each decade in response to the rapid development of drug resistance displayed by these pathogenic species^27,28^. In this paper we have quantified the binding of several azole drugs to both the WT and DM forms of the BM3 heme domain, demonstrating their much higher affinity for the DM protein. We also report the first structures of the BM3 DM heme domain in complex with four different azole drugs, confirming the greater plasticity of this variant and its ability to structurally adapt to bind to several azole drug compounds.

## Results

### Ligand binding assays of azole drugs to the WT and A82F/F87V (DM) BM3 heme domains

**Figure 1:**
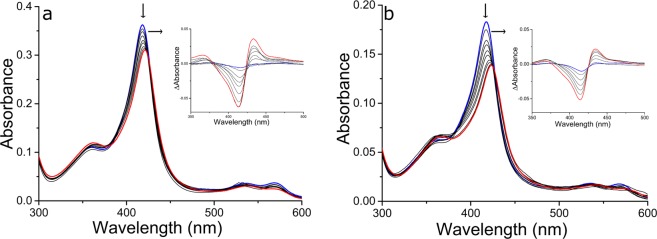
Binding of tioconazole to WT and DM BM3 heme domains. The main panels show spectral titrations of the WT BM3 heme domain (**a**) and of the DM BM3 heme domain (**b**) with tioconazole. In both cases, tioconazole binding induces a Soret red shift accompanied by decreased Soret intensity. The arrows indicate the direction of absorption change in each case. Tioconazole induces a more substantial Soret shift (from 418 nm to 423 nm) in the DM BM3 heme domain, and binds ~50-fold tighter to the DM heme domain than to the WT heme domain. The insets show difference spectra generated by subtracting each successive ligand-bound spectrum from that of the ligand-free heme domain. *K*_d_ values were determined by subtracting the absorbance value at the trough from that at the peak in each case (using the same wavelength pair throughout the titration) and by data fitting as described in the Methods section.

**Figure 2:**
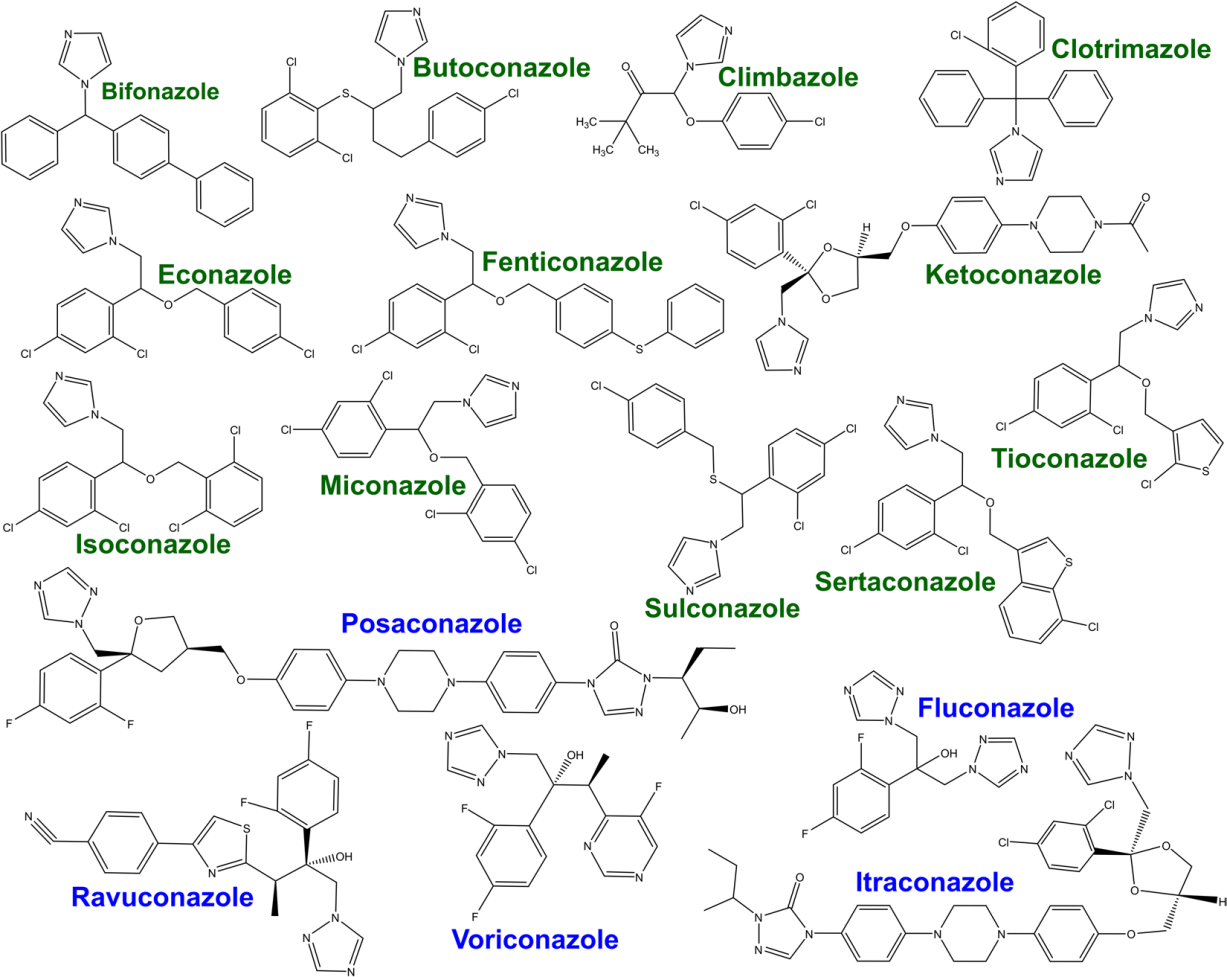
Structures of azole antifungal drugs used in binding studies with the P450 BM3 WT and DM heme domains. All the azoles shown are drugs that are currently (or were previously) marketed for the treatment of a variety of topical and/or systemic fungal infections. The binding of these compounds to the WT and A82F/F87V (DM) forms of the BM3 heme domain was analyzed using UV-visible spectroscopic titrations and by EPR analysis in a number of cases. Imidazoles are shown in green and triazoles are in blue.

In their resting, substrate-free forms, the WT and DM BM3 heme domains are in a predominantly low-spin ferric state with the heme iron axially coordinated by cysteine thiolate (proximal) and water (distal) ligands. The weakly bound distal water ligand is readily displaced by a substrate to produce a 5-coordinate high-spin (HS) ferric species that (in the case of the intact BM3 enzyme) is rapidly reduced to the ferrous state by an electron delivered from the CPR domain and obtained from NADPH. This leads to dioxygen (O_2_) binding to heme iron and to the progression of the P450 catalytic cycle that leads to monooxygenation of the substrate^29^. UV-visible spectroscopic titrations can be used to quantify the binding of substrates to intact P450 BM3 and its heme domain based on low-spin (LS) to HS type I spectral shifts (from ~418 nm to ~390 nm). The azole drugs described here all typically elicit a type II (red) Soret shift (mainly in the ~421 nm to ~425 nm range) that is indicative of the displacement of the heme iron axial water ligand by nitrogen ligation to the heme iron atom. As shown in Table 1, heme spectral shifts of different magnitudes occur according to the particular azole drug used, with more substantial Soret shifts typically observed for the BM3 DM heme domain on binding to azole inhibitor ligands (Fig. 1). P450 UV-visible binding assays and other spectroscopic studies using azoles are often hindered by the low solubility of many of these drugs. However, we were able to determine binding affinities by UV-visible spectroscopic titration for almost all of the azole drugs used, with both the WT and DM heme domains. Figure 2 shows structures of the 17 azole drugs that were used in these binding studies. The azoles used range widely in size and structure, and contain either an imidazole or a triazole functional group (or two triazole groups in the case of fluconazole). The triazoles typically elicit less substantial red shifts and produce higher *K*_d_ values for the WT and DM heme domains in comparison to the imidazole drugs. Spectral binding data were fitted using hyperbolic (Michaelis-Menten)^30^ or tight-binding (Morrison)^31^ equations, or by using the Hill function^32^ in cases where there was a clear sigmoidal dependence of heme absorbance change on azole drug concentration.

**Table 1:** Azole drug binding constants and associated spectral data for WT and DM BM3 heme domains.

Azole	MW (Da)	WT BM3				DM BM3			
		*K*_d_ (μM)	Soret Shift	Delta Abs Peak & Trough	Delta Abs/Soret Peak	*K*_d_ (μM)	Soret Shift	Delta Abs Peak & Trough	Delta Abs/Soret Peak
**Bifonazole**	310.4	3.54 ± 0.05	423 nm	434/413 nm	0.43	0.13 ± 0.05	425 nm	436/413 nm	0.42
**Butoconazole nitrate**	474.8	3.80 ± 0.12	422 nm	434/413 nm	0.35	0.47 ± 0.02	424 nm	433/413 nm	0.47
**Climbazole**	292.8	1.52 ± 0.02	424 nm	433/413 nm	0.43	0.32 ± 0.01	424 nm	430/408 nm	0.50
Clotrimazole*	344.8	32.8 ± 1.9	421 nm	435/414 nm	0.29	0.42 ± 0.02	423 nm	432/413 nm	0.41
**Econazole nitrate**	444.7	19.3 ± 1.1	421 nm	434/413 nm	0.27	0.53 ± 0.04	423 nm	435/414 nm	0.47
**Fenticonazole nitrate**	518.4	1.89 ± 0.05	419 nm	434/415 nm	0.21	0.52 ± 0.03	424 nm	434/414 nm	0.44
Fluconazole*	306.3	NB	—	—	—	2.91 ± 0.20	421 nm	434/414 nm	0.19
**Isoconazole nitrate**	479.1	12.0 ± 0.5	419 nm	436/415 nm	0.13	0.44 ± 0.02	422 nm	435/415 nm	0.37
*Itraconazole*	705.6	NB	—	—	—	0.05 ± 0.01	421 nm	438/414 nm	0.16
**Ketoconazole**	531.4	4.41 ± 0.15	423 nm	434/413 nm	0.38	0.50 ± 0.02	423 nm	433/414 nm	0.41
**Miconazole**	416.1	6.94 ± 0.15	421 nm	437/416 nm	0.37	0.63 ± 0.03	423 nm	433/413 nm	0.44
*Posaconazole*	700.8	NB	—	—	—	2.69 ± 0.05	422 nm	430/395 nm	0.41
*Ravuconazole*	437.5	NB	—	—	—	0.34 ± 0.02	422 nm	434/413 nm	0.21
**Sertaconazole nitrate**	500.8	2.44 ± 0.14	419 nm	439/416 nm	0.08	0.46 ± 0.01	423 nm	433/413 nm	0.45
**Sulconazole nitrate**	460.8	8.93 ± 0.54	421 nm	433/413 nm	0.28	0.52 ± 0.01	424 nm	434/413 nm	0.47
Tioconazole*	387.7	24.5 ± 1.1	422 nm	434/414 nm	0.29	0.39 ± 0.03	423 nm	434/414 nm	0.44
Voriconazole*	349.3	NB	—	—	—	1.90 ± 0.13	420 nm	435/414 nm	0.21

Several azole drugs were shown to bind to the WT and DM BM3 heme domains. Table 1 shows *K*_d_ values for each azole drug (NB indicates instances where no binding was seen in UV-visible spectroscopic titrations) and the final Soret wavelength shift observed (from a starting wavelength of 418 nm in each case) at apparent saturation with each azole. The Delta Abs Peak & Trough columns show wavelength maxima and minima from each absorbance difference spectrum. The Delta Abs/Soret Peak columns show values derived by dividing the maximal Soret peak-minus-trough values from each titration by the relevant P450 Soret peak value in each case (thus correcting for differences in P450 concentrations used in binding titrations). *K*_d_ values were determined as described in the Methods section, using either a hyperbolic function (Michaelis-Menten), the Hill function or the Morrison equation for tight-binding ligands, as appropriate. Bold text indicates imidazole drugs and italic text indicates triazole drugs. Azoles marked with an asterisk are those for which crystal structures of azole-bound complexes of the BM3 DM heme domain were solved. No significant heme absorbance changes were observed for the WT BM3 heme domain on titration with the triazole drugs.

The WT BM3 heme domain is able to bind many of the azole drugs. However, the DM heme domain binds these azoles far more tightly, and much greater heme spectral shifts are induced on their binding to the DM heme domain. This is a result of the combined structural rearrangements that are induced by the A82F mutation (resulting in an enlarged active site) and by the F87A mutation (where removal of the Phe87 phenyl side chain creates further binding space close to the heme). Examples of azole drug binding titrations are shown in Fig. 1 for the imidazole drug tioconazole with WT and DM heme domains. The binding affinities for the WT and DM heme domains are 18.4 ± 0.9 μM and 0.39 ± 0.03 μM, respectively. Table 1 shows the collated data sets for the binding of the 17 different azoles to WT and DM heme domains, including spectral shifts observed, *K*_d_ values for azole binding, and the binding model used (Michaelis-Menten, Hill or Morrison equations). Examples of each type of binding plot are presented in Figure S1. Only in the cases of the five triazole drugs with the WT heme domain were negligible changes in the heme spectrum observed.

### Electron paramagnetic resonance (EPR) studies of WT and A82F/F87V (DM) BM3 heme domains bound to azole drugs

**Figure 3:**
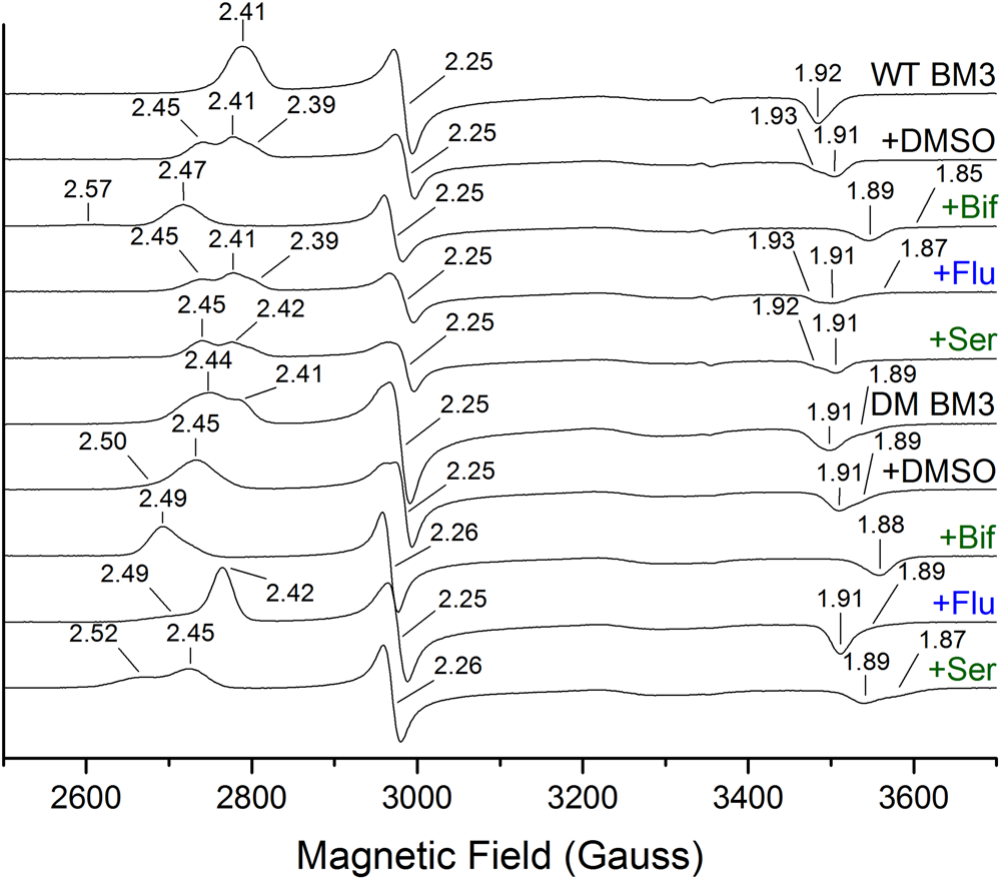
X-band EPR spectra for the WT and DM BM3 heme domain complexes with azole drugs. Selected spectra for the WT and DM BM3 heme domains are shown in (i) the azole drug-free state, (ii) in presence of DMSO (5% v/v), and (iii) in complex with the azole drugs bifonazole, fluconazole and sertaconazole (with 5% DMSO). The g-values are indicated in each spectrum, and the EPR data for these and other species are presented in Table S2 and Table S3. Spectra were recorded at 10K with a microwave power of 2.08 mW and modulation amplitude of 1 mT. DMSO was used to solubilize the azole drugs, but can itself also interact with heme iron in these enzymes at high concentrations. However, in UV-visible ligand binding titrations DMSO levels did not exceed 0.4% v/v.

X-band EPR was used to analyze the binding of azole drugs to the WT and DM BM3 heme domains in their ferric state. There was no significant HS component seen in any of the EPR spectra, consistent with the known properties of the BM3 WT and DM heme domains in their resting hexacoordinated states, and with the low temperature required for EPR spectroscopic analysis of ferric hemes (10 K in this case)^33^. The azole drugs are strong inhibitors that either displace the water ligand to coordinate the heme iron directly through nitrogen atoms from imidazole or triazole rings, or in some cases may instead bind indirectly through a retained distal water ligand^24^. The WT, ligand-free heme domain shows a single EPR species with g-values of g_z_ = 2.40, g_y_ = 2.25, g_x_ = 1.92 (2.40/2.25/1.92), while the DM heme domain shows some heterogeneity with two sets of g-values at 2.44/2.25/1.89 and 2.41/2.25/1.91. The heterogeneity likely arises due to the greater conformational flexibility of the DM heme domain, for which the ligand-free A82F X-ray crystal structure (PDB 4KF0) revealed a P450 conformation similar to that for a substrate-bound form of the WT heme domain^22^. Dimethyl sulfoxide (DMSO) was used as a solvent for the azole drugs and was found to influence the EPR spectra at high concentrations, likely through interactions made with the heme iron through its sulfur atom^34^. Figure 3 shows overlaid EPR spectra for the WT and DM heme domains in the ligand-free state and when bound to various azole ligands, with g-values shown. In general, the imidazole drugs induce more substantial changes to the EPR spectrum than do the triazoles for the WT heme domain, although fluconazole and posaconazole both produce significant shifts in the g-values for the DM heme domain. DMSO binding to the WT heme domain results in a triplet set of g-values, whereas its binding to the DM heme domain produces a homogeneous EPR spectrum. As observed in the spectral binding assays, no clear evidence was obtained from EPR that was indicative of triazole drug binding to the WT heme domain. The EPR spectral changes seen in the presence of the triazoles were essentially the same as those observed with DMSO alone.

### X-ray crystallography of azole-bound DM heme domain complexes

Crystal structures were obtained for the DM BM3 heme domain bound to clotrimazole (PDB 6H1T), fluconazole (PDB 6H1S), tioconazole (PDB 6H1L) and voriconazole (PDB 6H1O). Voriconazole^35^, clotrimazole^36^ and fluconazole^37^ have previously been crystallized in complex with eukaryotic P450s. Structures of fluconazole complexes with *M*. *tuberculosis* CYP121A1 and CYP51B1 enzymes have also been solved^24,37^. However, the structure of a P450 bound to tioconazole has not been reported previously. In order to produce the DM heme domain complexes, the protein was co-crystallized with 5 mM ligand (added in a DMSO stock). Structures of DMSO-bound P450 BM3 heme domain crystals have been reported previously^34^. However, no DMSO was observed in any of the structures reported in this study. All crystals diffracted to a resolution of ~2.2–1.8 Å. A table of crystallographic data for the four DM heme domain azole-bound structures, together with conditions used to obtain the crystal structures, is shown as Table S1.

Previous studies revealed the binding of an imidazole ligand to the DM heme iron with a bond distance of 1.80 Å (PDB 4KF2)^22^. Each of the novel structures presented in this study also show the direct ligation of a hetero-aromatic nitrogen atom to the DM heme iron with slightly longer bond lengths than those in the imidazole-bound form (ranging from 2–2.2 Å). However, a novel ligand binding mode was observed for voriconazole (see below). For all the azole-bound structures reported in this study, the hetero-aromatic nitrogen ligand is oriented so as to fit within the groove of the I-helix formed between Ala264 and Glu267/Thr268. While this places the distinct ligands in the same relative position, large differences occur in the nature and orientation of the various non-heme ligating substituents. As a result, despite a similar overall protein conformation, the positioning of the various hydrophobic amino acid side chains is dependent on the nature of the ligand. Furthermore, the conformation of the C-terminal loop protruding into the active site, containing Leu437, is also dependent on the particular ligand. Compared to the 4KF2 structure, residue Leu437 occupies an area slightly further from the ligand for all the azole-bound structures reported in this manuscript. In contrast, Ala264 and Thr268 of the I-helix align very closely in all structures, including 4KF2. Glu267 exhibits the greatest change between structures. This residue aligns closely for the 4KF2 structure and the tioconazole-bound structures, but changes in Glu267 conformation are observed for the fluconazole- and voriconazole-bound structures, and substantial changes are seen in the clotrimazole-bound structure.

The voriconazole, clotrimazole and tioconazole structures contain multiple monomers in the asymmetric unit, and in each case the general protein conformation corresponds to the “open”, substrate-free conformation of the enzyme, with little significant difference in protein or ligand conformation between monomers. In contrast, the fluconazole complex contains two distinct monomer forms in terms of their conformation; one corresponding to the “open” form, and a second monomer where the F/G helices (which are important structural elements controlling active site substrate access and product egress) have reoriented to resemble a “closed” P450 conformation. As a consequence, two distinct orientations are observed for the bound fluconazole ligand. Although fluconazole does not interact directly with the mutated residue Phe82, the aromatic side chain of this residue was observed to undergo a ~90° rotation relative to that seen in the 4KF2 and the other azole-bound structures reported here. Fluconazole is the only one of the four azole drugs studied that does not occupy space close to Phe82, enabling this residue to rotate in this way. However, in 4KF2 the Phe82 residue also has the space to rotate in this way, but does not rotate its side chain in the manner observed for the fluconazole-bound DM heme domain.

**Figure 4:**
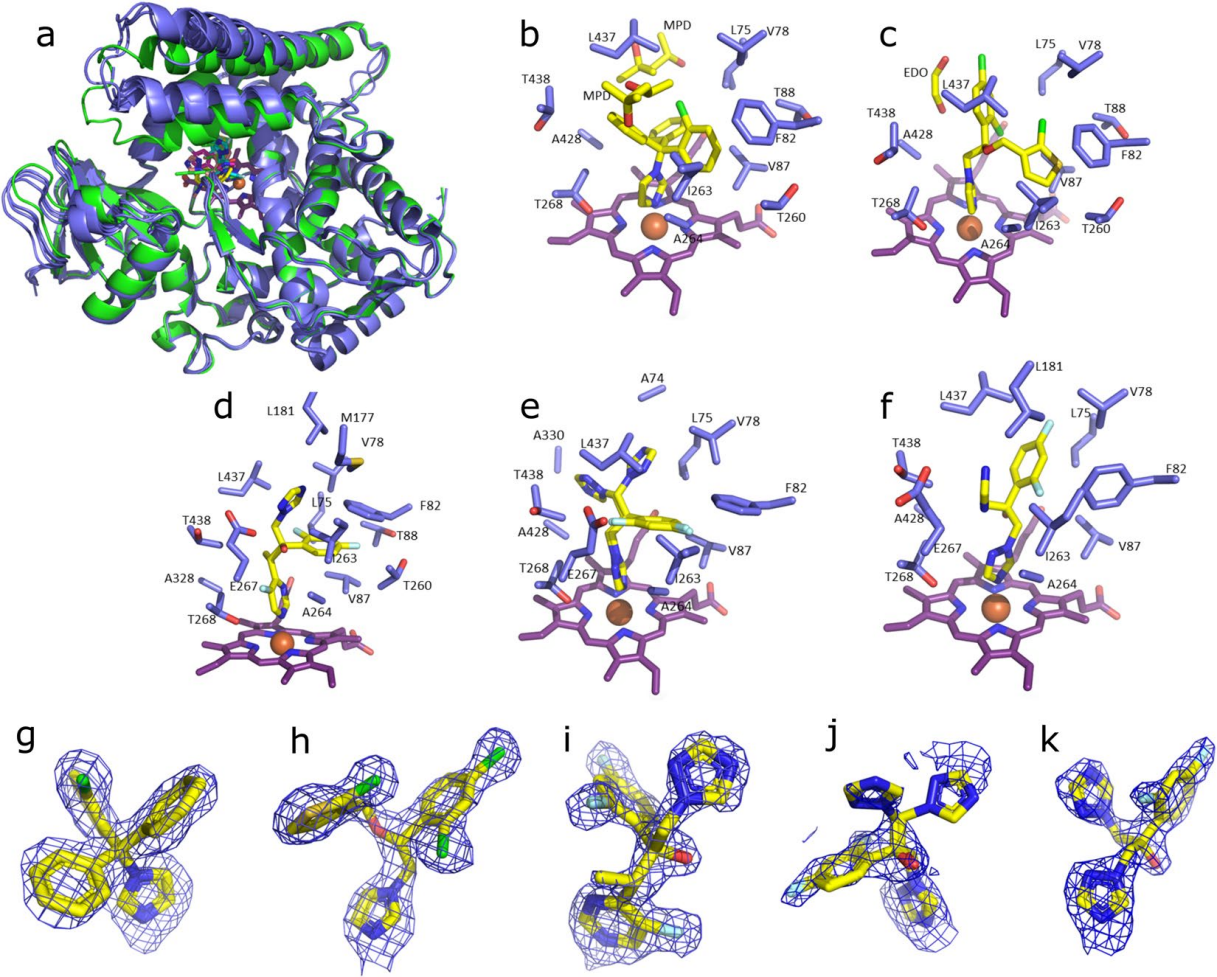
Crystal structures of the BM3 heme domain A82F/F87V mutants in complex with different azole drugs. Panel a shows an overlay of various azole-complexed A82F/F87V (DM) BM3 heme domain structures, revealing that a similar overall conformation (“open”, in blue) is adopted by the majority of the monomers. In contrast, a single monomer in the fluconazole complex is in a “closed” stated (shown in green). Panels b-f depict the environment of the bound ligands (clotrimazole - b, tioconazole - c, voriconazole - d and fluconazole - e/f, respectively), with key residues shown in atom colored sticks (ligand with yellow carbons, protein with light blue carbons and heme with purple carbons). Panels g-k depict the corresponding omit electron density (contoured at 3 sigma) for each of the ligands (clotrimazole - g, tioconazole - h, voriconazole - i and fluconazole - j/k) as a blue mesh. With the exception of fluconazole, the conformation of the ligand is similar in the various monomers in the asymmetric unit, and in each instance monomer A is shown. In the case of the fluconazole ligand, both of the conformations observed are depicted in panels e (“open” conformation) and f (“closed” conformation), with the corresponding electron density shown in panels j and k. MPD: 2-methyl-2,4-pentanediol, EDO: 1,2-ethanediol.

Figure 4 shows an overlay of the four crystal structures of the ligand-bound DM heme domain, together with images of the active sites of the DM heme domain in their complexes with clotrimazole, tioconazole, fluconazole and voriconazole. In the case of voriconazole, it was clear the *2 R*,*3R*-stereoisomer was bound, while for tioconazole the *R-*tioconazole was identified as the bound stereoisomer. With the exception of the heme-ligand interaction, most contacts established with the protein are of a hydrophobic nature. In the case of clotrimazole, one of the most hydrophobic ligands, two additional 2-methyl-2,4-pentanediol (MPD) molecules derived from the mother liquor were observed to interact both with the ligand and the protein. As binding titrations and EPR studies were conducted without MPD or similar compounds, MPD is clearly not needed for azole binding. Similarly, small mother liquor-derived compounds are present in the case of tioconazole (a molecule of 1,2-ethanediol) and voriconazole (a molecule of glycerol). One of the few direct polar interactions observed between the protein and one of the azole ligands is made between the triazole moiety of voriconazole and Glu267. Surprisingly, voriconazole coordinates the heme iron through a pyrimidine nitrogen from its 5-fluoropyrimidine ring. All other voriconazole-bound P450 crystal structures on the PDB show coordination of the P450 heme iron through a triazole nitrogen, and thus this is a unique binding mode for voriconazole^35,38–40^. No direct polar interactions are observed for fluconazole, the only other ligand with a free triazole group. For fluconazole, two different conformations are observed, linked to the distinct conformations of the DM heme domain protein itself. In the “open” structure, the electron density of the bound fluconazole is relatively poor, indicating multiple conformations for the non-heme bound triazole group. In contrast, the “closed” monomer contains a well-defined fluconazole ligand that does not display significant conformational heterogeneity. In comparison, the voriconazole, clotrimazole and tioconazole structures contain multiple monomers in the asymmetric unit, and in each case the general protein conformation corresponds to the “open”, substrate-free conformation of the enzyme, with little significant difference in protein or ligand conformation between monomers.

## Discussion

Azole drugs were developed in the 1960s and have been used widely as inhibitors of fungal 14α-lanosterol demethylases (CYP51 family enzymes). The azole drug-mediated inhibition of sterol demethylation in fungi ultimately prevents the formation of ergosterol, which is a key regulator of membrane fluidity and permeability^41^. The first azole drugs (clotrimazole, econazole and miconazole) developed in the 1960s had unacceptable side effects when administered orally^42^. This stimulated the development of safer azoles such as ketoconazole, which became the industry leading azole until triazoles and advanced imidazole drugs were developed^43^. For example, the antifungal drug clotrimazole is known to inhibit many human P450s, but newer generations of azole drugs, such as voriconazole, appear to inhibit fewer human P450s^44^. While azoles are best known for their antifungal activity in CYP51 enzymes, they have also been used widely as inhibitors of other eukaryotic and prokaryotic P450 enzymes. For example, the crystal structure of human CYP46A1 was solved in voriconazole- and clotrimazole-bound forms^35^, as well as in a posaconazole-bound state^27^. The structures of the *Mycobacterium tuberculosis* sterol demethylase CYP51B1 bound to fluconazole^37^ and the *M*. *tuberculosis* cyclodipeptide oxidase CYP121A1 bound to fluconazole have also been determined^24^.

Previous studies of the WT P450 BM3 identified the binding of small imidazole-based compounds (e.g. 1-phenylimidazole and 4-phenylimidazole), and in other work we have reported the binding of omega-imidazolyl carboxylic acids (C10-C12) to the WT P450 BM3 with *K*_d_ values in the range from ~0.2 μM to 27 μM^45^. In this paper we present a systematic study of the binding of 17 different azole antifungal drugs to both (i) the WT heme domain and (ii) a double mutant (DM, A82F/F87V) form of the heme domain in which these mutations confer greater conformational flexibility on the P450, in addition to generating more space in the active site cavity of the enzyme. Table 1 shows that the WT BM3 heme domain is able to bind to several different azole drugs that are in clinical use. In so doing, the azoles ligate to the ferric heme iron of the WT heme domain through nitrogen atoms. This occurs through imidazole nitrogens for clotrimazole/tioconazole, and through a triazole nitrogen for fluconazole. A novel DM heme iron ligation occurs for voriconazole, through a pyrimidine nitrogen on the voriconazole 5-fluoropyrimidine ring. The azole *K*_d_ values for the WT heme domain ranged from 1.89 μM (fenticonazole nitrate) to 32.8 μM (clotrimazole). All five triazoles tested on the WT heme domain (itraconazole, posaconazole, fluconazole, voriconazole and ravuconazole) failed to induce a Soret spectral shift of sufficient extent to allow *K*_d_ determination. For the DM heme domain, all 17 azoles tested produced type II heme Soret spectral shifts, with *K*_d_ values ranging from 0.05 μM (itraconazole) to 4.53 μM (fluconazole). In general, the azole binding-induced Soret band (red, type II) spectral shifts for the DM heme domain are more substantial than those for the WT heme domain (by ~2 nm on average), discounting those azoles that failed to induce any significant Soret spectral shifts in the WT heme domain.

The crystal structures of the BM3 DM heme domain were solved in their complexes with the azoles clotrimazole, voriconazole, fluconazole and tioconazole (Fig. 4). It is clear that the conformational flexibility of the A82F/F87V DM variant is an important property of the enzyme that facilitates the full binding of these ligands and enables the structural analysis of their crystallized complexes. In contrast, the WT heme domain is less flexible and typically displays less extensive spectral shifts on binding to these azoles, and no significant spectral shifts for itraconazole, posaconazole and ravuconazole. Several of the azoles used in this study showed apparent binding cooperativity (i.e. a sigmoidal dependence of spectral change on azole drug concentration) in their binding to WT and/or DM heme domains (e.g. with butoconazole nitrate and ketoconazole for both WT/DM heme domains). However, X-ray crystallographic analysis revealed single occupancy of the azole ligands for each of the structures solved. In EPR studies, many of the azole drugs used display heterogeneity (i.e. two or more sets of g-values). However, for azole ligands such heterogeneity can arise as a result of e.g. the presence of a proportion of the P450 heme iron with water as the distal axial ligand, and one or more other species in which an azole nitrogen ligates directly to the heme iron, or makes an interaction through the distal water ligand, as was observed for the binding of fluconazole to the heme iron of the *M*. *tuberculosis* cyclic dipeptide oxidase enzyme CYP121A1^24^. The binding of three of the azole drugs induced a minor “narrowing” of the DM heme domain EPR spectra compared to that for the azole-free form (g-values of 2.44/2.25/1.91). These were for the major species observed for ketoconazole (2.43/2.26/1.90), posaconazole (2.42/2.26/1.91) and fluconazole (2.42/2.25/1.91). However, in each case a further species was formed in which the relevant azole drugs ligate to the heme iron (2.53/2.26/1.87, 2.50/2.26/1.89 and 2.49/2.25/1.89, respectively). Complete sets of EPR data for ligand-free and ligand (azole drug)-bound forms of the WT and DM heme domains are presented in Tables S2 and S3, respectively).

In previous work, P450 BM3 heme domains were co-crystallized with 14% DMSO (F87A) or 28% DMSO (WT and F87A)^46^. It was hypothesized that the DMSO sulfur atom competes with the distal water molecule. This is consistent with our EPR studies, which show that the WT heme domain X-band EPR spectra become more heterogeneous in the presence of DMSO. That is, DMSO-bound and water-ligated states can co-exist in the samples. However, our DM heme domain samples become more homogeneous in presence of DMSO, possibly due to DMSO displacing the distal water and interacting more effectively with the DM heme iron. DMSO effects are also seen in collision-induced unfolding (CIU), different forms of mass spectrometry and other methods^47^. However, no DMSO molecules were observed in any of the four crystal structures solved. DMSO added in isolation causes a small Soret red shift on binding to the ligand-free DM BM3 heme domain. However, no significant absorbance change is observed for the same DMSO addition to the WT BM3 heme domain. The binding of azole drugs displaces the DMSO as an axial ligand due to their greater size and higher affinity for the heme iron. In the EPR studies presented in this paper – DMSO in isolation is added at 5% v/v to demonstrate that DMSO can bind effectively to heme iron if added in large amounts. Much smaller amounts of DMSO (less than 0.4% v/v) were used for azole binding titrations, in which the azole drugs bind preferentially to the heme iron.

The azole-bound DM heme domain structures solved in this study all contain a single molecule of their respective azoles, although chemicals from the relevant mother liquor (glycerol, 1,2-ethanediol and 2-methyl-2,4-pentanediol) are also observed in the active sites. However, the DM has a large active site volume and could potentially interact with a second molecule of the ligand. Indeed, recent structural studies with another P450 BM3 steroid-oxidizing mutant enzyme revealed the binding of two molecules of testosterone, one at the catalytic site, with the second binding closer to the active site entry region^48^. In solution state ligand-binding titrations, several of the azole drugs produced sigmoidal binding curves indicative of cooperative binding (Table 1). This could occur through e.g. co-binding of two ligands to the heme domain, or by interactions between heme domains. However, the WT BM3 heme domain is monomeric in solution. Many eukaryotic P450 enzymes exhibit cooperativity in ligand binding. A good example is seen in the case of the major human drug-metabolizing protein CYP3A4. An allosteric binding site was identified on CYP3A4 which contributes to the cooperativity observed for both substrate and inhibitor binding^49^. WT BM3 was also reported to show chain length-dependent cooperativity with fatty acids^50^.

A number of residues were identified from our azole-bound structures which appear crucial to azole binding: Ala264, Glu267, Thr268, and Leu437. These residues are close to the heme prosthetic group and allow both electrostatic and hydrogen bonding interactions to occur with the ligand. Glu267 and Thr268 are important for oxygen binding and activation^51^. Thr268 is also implicated in substrate recognition^52^, although the role of Leu437 is less clear. However, a hydrogen bond was observed between Leu437 and the FDA-approved drug omeprazole in the structure of the A82F heme domain variant of BM3^22^. Ala264 also occupies a position close to the heme prosthetic group in BM3. This residue aligns with a conserved glutamate that can form an ester bond to a heme methyl group in mammalian family 4 P450 enzymes^53^. CYP4 family enzymes, like P450 BM3, can bind a variety of fatty acid substrates (e.g. lauric acid and arachidonic acid), as well as steroids (e.g. testosterone) in humans^44^.

## Conclusions

In this paper, we have investigated (i) structural (X-ray crystallography), (ii) spectroscopic (EPR) and (iii) binding affinity (*K*_d_) properties of the WT and DM (A82F/F87V) P450 BM3 heme domains. Each of these methods provides its own important insights into (i) the mode(s) of binding of azole drugs to the DM mutant, (ii) the heterogeneous nature of azole ligand binding, and (iii) the affinity of the azoles used for the WT and DM heme domains. The azole drugs are used widely as antifungal medications. However, many pathogenic fungi are becoming resistant to current antifungal drugs, including several drugs of the azole class^26^. Paths to combat this drug resistance may be to improve current azoles (and other antifungals) by increasing their bioavailability, reducing side effects and improving the spectrum of these drugs^54^. The P450 BM3 enzyme has long served as a model system for eukaryotic P450s, in view of its soluble nature and its natural interaction with a (fused) eukaryotic-like cytochrome P450 reductase enzyme^55^. There has been no previous report of a P450 BM3 crystal structure in complex with an azole antifungal drug. Our current crystallographic study demonstrates how the structurally altered A82F/F87V variant of the P450 BM3 heme domain binds to four different azole drugs: two imidazoles (clotrimazole and tioconazole) and two triazoles (fluconazole and voriconazole). Key residues interacting with the azoles in these structures are Ala264, Glu267, Thr268, and Leu437. In previous crystallographic studies with the *M*. *tuberculosis* CYP121A1 P450, the major binding mode of fluconazole was observed to occur through its interaction with a retained distal water molecule on the heme iron^24^. However, fluconazole coordinates directly to the DM BM3 heme iron in the crystal state, as is also the case for its interaction with the *M*. *tuberculosis* sterol demethylase enzyme (CYP51B1)^37^. The crystal structure of tioconazole-bound DM heme domain is the first shown to bind this azole drug. The binding mode of tioconazole to the DM heme domain is similar to those of clotrimazole and voriconazole, though the latter interacts with the heme prosthetic group through a novel ligation via a pyrimidine nitrogen from its 5-fluoropyrimidine ring. However, fluconazole exhibits a different binding mode as the protein exists in the “closed” conformation with more extensive active site occupancy of the fluconazole ligand. In addition, fluconazole does not approach closely to the key conformation altering mutant residue (Phe82), allowing the phenyl side chain of this residue to rotate by ~90°.

In azole drug ligand-binding studies, numerous azoles of diverse size and structure were found to bind to both the WT and the DM heme domains. EPR was also used to explore azole drug binding, although the DMSO used to solubilize the azoles may compete to a small extent for heme iron binding in some instances. In all cases, the *K*_d_ values determined by optical titrations for the DM were significantly lower than those for the WT heme domain, demonstrating that the F87V and A82F mutations are conducive to conformational reorganization in the DM heme domain that facilitates the improved binding of all of the azoles relative to their *K*_d_ values in the WT heme domain. In addition, there is a larger azole-induced DM Soret spectral shift, which in turn is indicative of a greater extent of azole ligand binding to the DM heme iron. The greater level of azole drug occupancy achieved with the DM mutant heme domain clearly proved crucial for the successful crystallization and structural determination of the four different azole drug complexes of the DM heme domain. Other studies with the DM heme domain also enabled the determination of the first crystal structure of BM3 in complex with a human drug substrate (the gastric proton pump inhibitor omeprazole)^22^. Very few structures are available to date for azole antifungal drugs in complex with human P450s. These are (i) the cholesterol hydroxylase CYP46A1 (bound to clotrimazole, voriconazole and posaconazole), which is located within the brain and eyes^27,35^; and (ii) ketoconazole bound to the major human drug-metabolizing P450 CYP3A4^56^ and to the human lanosterol 14α-demethylase (CYP51)^57^. As a result, the BM3 DM heme domain (or variants thereof) could have important applications in analysis of the binding modes of other azole drugs. Novel insights gained would include understanding the types of molecular interactions that are favored by specific azole drug substituent groups, as well as establishing if indirect azole nitrogen binding to a retained distal water on the heme iron can also occur in BM3 mutants.

## Methods

### Expression and purification of WT and A82F/F87V (DM) BM3 heme domain proteins

The BM3 heme domain proteins (WT and the DM A82F/F87V variant) used in this study were expressed as described in our previous studies^53,58^. Delta-aminolevulinic acid (0.1 mM) was added to the *E*. *coli* BL21 (DE3) transformant culture to promote heme incorporation in the case of the DM heme domain when the OD_600_ of the culture reached 0.6. Cells were pelleted by centrifugation at 4 °C (6000 g, 20 min). The cells were resuspended in a small volume of ice-cold buffer A (50 mM Tris plus 1 mM EDTA, pH 7.2). Protease inhibitors (two EDTA-free cOmplete^TM^ tablets, Roche Applied Science), MgCl_2_ and DNase (10 μg mL^−1^) were added to the mixture and the cell suspension was lysed by sonication on ice using a Bandelin Sonopuls sonicator at 40% power, with 12 pulses for 40 s and with 60 s between pulses). The supernatants containing the WT and DM heme domains were separated from the cell debris by centrifugation (46,000 g, 60 min, 4 °C). Ammonium sulfate was added to 30% saturation at 4 °C with slow stirring for ∼1 h to precipitate contaminant proteins. The sample was then centrifuged again (46,000 g, 15 min, 4 °C) to remove cell debris.

The WT and DM BM3 heme domain-containing suspensions were further purified on a 150 mL DEAE Sepharose™ fast flow resin (GE Healthcare Life Sciences) after buffer exchange by dialysis into buffer A. A linear gradient of 0–300 mM KCl in buffer A (1500 mL) was applied and the fractions containing the WT and DM heme domains (both red in color) were further purified by loading onto a 28 mL CHT hydroxyapatite type 1 resin (Bio-Rad) column in 25 mM potassium phosphate (KPi) buffer (pH 6.5). A linear gradient of 25–300 mM KPi (600 mL) was applied to fractionate the heme proteins. For protein samples destined for X-ray crystallography, a further purification step was used. A Q-Sepharose™ Fast Flow column was used with a gradient of 0–300 mM KCl in 50 mM KPi (pH 7.2) (1000 mL), followed by further resolution on a HiLoad™ GF S200 16/600 Superdex™ 200 pg column (GE Healthcare Life Sciences) using a 25 mM KPi (pH 7) buffer containing 150 mM NaCl. Protein purity was determined by SDS-PAGE analysis and the Reinheitszahl (Rz) purity ratios (A_418_/A_280_) were determined by UV-Vis spectroscopy^59,60^. For X-ray crystallography, heme domain samples with Rz values over 1.5 were used. For other experiments (e.g. ligand binding), heme domain samples with Rz values of at least 1.3 were used. P450 BM3 naturally binds fatty acids, such as those found in *E*. *coli* during expression of the protein. As a result P450 BM3 is typically found in a mixed–spin state (combination of low-spin and high-spin states) after expression and purification. Lipids were removed from the proteins by passing the heme domains through a 20 mL Lipidex™-1000 column in 25 mM KPi (pH 7).

### UV-Visible spectroscopic assays of azole drug binding to WT and DM BM3 heme domains

Experiments were performed using a Cary 50 UV-Visible spectrophotometer (Agilent Technologies Ltd). WT and DM heme domains at concentrations of ~2–4 μM were used, and binding titrations were done by the addition of small (typically 0.2–0.4 μL) volumes of azole ligand stocks (1, 10 or 30 mM, to minimize amounts of solvent carrier used) to a 1 mL heme domain sample in a quartz cuvette. An extinction coefficient of 95 mM^−1^ cm^−1^ was used for the low-spin forms of the WT and DM heme domains. Hamilton syringes were used to deliver azole drugs samples until no further spectral shifts were observed. Azole drugs were obtained from Merck Chemicals (Nottingham, UK). All azole compounds used in these and other experiments done in this manuscript were sourced from Sigma Aldrich (Cambridge, UK). All compounds used were racemic mixtures, apart from clotrimazole, fluconazole and posaconazole.

Data were processed by the subtraction of the spectrum of each successive ligand-bound form (from a specific titration set) from the spectrum of the ligand-free enzyme. This produced a set of difference spectra (for each particular azole titration), from which wavelengths associated with the absorbance minimum (A_min_) and maximum (A_max_) were readily identified. For each titration point, an absorbance difference value was calculated as A_max_ minus A_min_, using the same wavelength pair throughout an individual experiment. These values were then plotted against the relevant azole ligand concentrations used. Data were fitted using either the Michaelis-Menten (hyperbolic) function, the Morrison equation (for tight binding substrates where the *K*_d_ value is ≤5x the P450 concentration) or the Hill equation for sigmoidal curves to determine *K*_d_ values^31,61,62^. Examples of each type of binding plot are presented in Figure S1. Assays were done using 25 mM KPi (pH 7) at a constant temperature of 30 °C.

### EPR spectroscopy studies of WT and DM BM3 heme domains bound to azole drugs

Samples of ligand-bound protein were prepared by incubation overnight at 4 °C with agitation at 10 rpm. Samples contained 200 μM protein in 25 mM KPi (pH 7) with the addition of 5 mM azole drug in DMSO, or appropriate volumes of DMSO or buffer as controls. X-band EPR spectra were recorded on a Bruker ER-300D series electromagnet with a microwave source interfaced with a Bruker EMX control unit and fitted with an ESR-9 liquid helium flow cryostat (Oxford Instruments), and a dual-mode microwave cavity from Bruker (ER-4116DM). Spectra were recorded at 10 K, microwave power was 0.5 mW, modulation frequency was 100 KHz and the modulation amplitude was 5 G.

### X-ray crystallography of the DM BM3 heme domain with azole drugs

Samples of azole ligand-bound protein were prepared with 9 mg mL^−1^ protein and 5 mM ligand (dissolved in a 100% DMSO stock) in 25 mM KPi (pH 7), followed by an overnight incubation at 4 °C with mixing at 10 rpm. Any precipitate was removed by centrifugation at 14,000 rpm for ten minutes in a microfuge. Crystallography was performed using the sitting drop method at 4 °C using Molecular Dimensions screening plates (PACT, SG1, MORP, JCSG+, BCS, Midas, LMB). 300 nL of the DM heme domain were added to plates using a Mosquito liquid handling robot (TTP LabTech Ltd). For all structures determined, seeding was used to improve crystal diffraction quality. Seeding was implemented to improve crystal size and quality using Molecular Dimensions screening plates. In these cases, 250 nL of the DM heme domain (prepared as above) were mixed with 50 nL seed stock and 300 nL mother liquor. Data were collected at Diamond synchrotron beamlines and reduced and scaled using XDS (28). Structures were solved and refined by molecular replacement with a previously solved BM3 heme domain structure (PDB 4KF2) using Phenix^63^, PDB re-do^64^ and Coot^65^_._ Crystallographic data and conditions used for the successful crystallization for each azole-bound crystal are presented in Table S1.

## Supplementary information

Supplementary Information
