# Supplementary material for: Novel insights into P450 BM3 interactions with FDA-approved antifungal azole drugs
Source: Sci Rep. 2019 Feb 7;9:1577. doi: 10.1038/s41598-018-37330-y (PMC6367340; doi:10.1038/s41598-018-37330-y)
Supplement: Supplementary file 1 — Supplementary Information [file 41598_2018_37330_MOESM1_ESM.pdf]

Novel insights into P450 BM3 interactions with FDA-approved antifungal azole drugs

Laura N. Jeffreys[1], Harshwardhan Poddar[1], Marina Golovanova[1], Colin W. Levy[2], Hazel M. Girvan[1], Kirsty J. McLean[1], Michael W. Voice[3], David Leys[1], and Andrew W. Munro[1,*]

[1]Centre for Synthetic Biology of Fine and Specialty Chemicals (SYNBIOCHEM), Manchester Institute of Biotechnology, School of Chemistry, The University of Manchester, Manchester M1 7DN, United Kingdom
[2]Manchester Protein Structure Facility (MPSF), Manchester Institute of Biotechnology, School of Chemistry, The University of Manchester, Manchester M1 7DN, United Kingdom
[3]Cypex Ltd., 6 Tom McDonald Avenue, Dundee DD2 1NH, Scotland, United Kingdom
[*]Correspondence and requests for materials should be addressed to A.W.M. (email: andrew.munro@manchester.ac.uk)

SUPPLEMENTARY INFORMATION

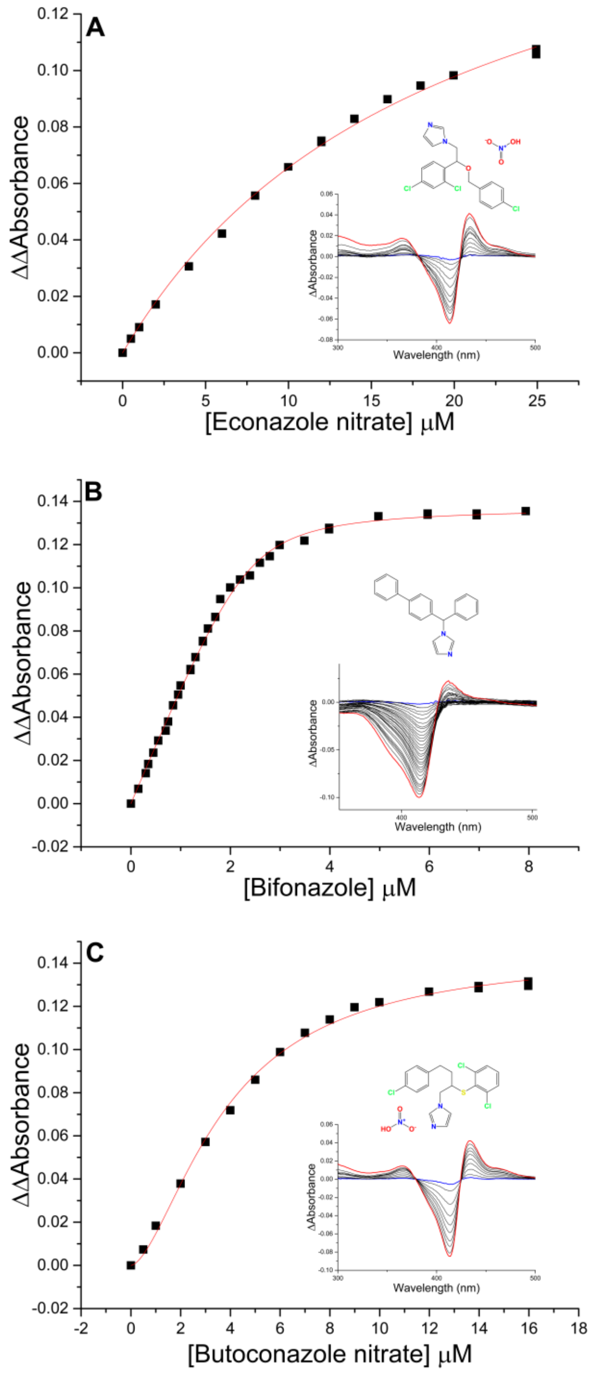

Figure S1: Azole drug binding plots for econazole, climbazole and butoconazole. Plots A-C show data sets for the binding of econazole, bifonazole and butoconazole to BM3 heme domains. Spectral titrations and data fitting were done as described in the "*UV-Visible spectroscopic assays of azole drug binding to WT and DM BM3 heme domains*" section. **Panel A** shows binding data for econazole with the WT enzyme fitted using a hyperbolic (Michaelis-Menten) equation to give a K_d value of 19.3 ± 1.1 μM. **Panel B** shows binding data for bifonazole with the DM enzyme fitted using the tight-binding (Morrison) equation to give a K_d value of 0.13 ± 0.02 μM. **Panel C** shows binding data for butoconazole with the WT enzyme fitted using a sigmoidal (Hill) equation to give a K_d value of 3.80 ± 0.12 μM. In each case, the UV-visible difference spectra induced by azole drug binding in the P450 Soret peak region are shown as an inset, along with the structure of the relevant azole drug.

	Clotrimazole 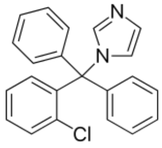	Fluconazole 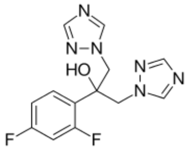	Tioconazole 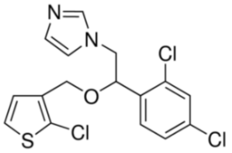	Voriconazole 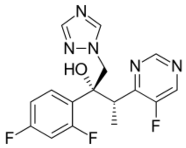
**PDB ID**	**6H1T**	**6H1S**	**6H1L**	**6H1O**
**Data Collection**				
Space group	$P2_1$	$P2_12_12_1$	$P2_1$	$P2_1$
Cell dimensions				
a, b, c (Å)	79.0, 70.8, 209.6	60.9, 119.3, 146.5	59.0, 150.9, 61.0	59.8, 149.9, 61.0
α, β, γ (°)	90.0, 95.3, 90.0	90.0, 90.0, 90.0	90.0, 95.7, 90.0	90.0, 96.8, 90.0
Resolution range (Å)	208-2.08 (2.12-2.08)	119.29-1.95 (1.99-1.95)	60.96-1.97 (2.00-1.97)	60.63-1.73 (1.76-1.73)
R_{meas}	0.080 (0.591)	0.113 (0.815)	0.047 (0.533)	0.088 (0.383)
CC1/2<br>$I/\sigma I$	1.0 (0.8)<br>11.8 (2.2)	1.0 (0.8)<br>10.7 (2.1)	1.0 (0.8)<br>15.2 (2.2)	1.0 (0.9)<br>8.2 (2.2)
Completeness (%)	91.7 (98.9)	100.0 (100.0)	98.6 (99.9)	90.6 (93.8)
Redundancy	3.4 (3.4)	6.6 (6.1)	3.4 (3.5)	3.0 (2.8)
**Refinement**				
Resolution (Å)	2.08	1.95	1.97	1.73
R_{work} / R_{free}	0.170/0.213	0.162/0.200	0.189/0.215	0.159/0.199
R.m.s. deviations				
Bond lengths (Å)	0.007	0.010	0.005	0.012
Bond angles (°)	0.963	1.007	0.754	1.265
Ramanchandran				
Favoured (%)	97.1	96.4	95.4	96.5
Outlier (%)	0.3	0.6	0.8	0.3
Crystallographic conditions	0.12 M Monosaccharides mix (0.2 M D-glucose; 0.2 M D-mannose; 0.2 M D-galactose; 0.2 M L-fucose; 0.2 M D-xylose; 0.2 M N-acetyl-D-glucosamine), 0.1 M Buffer System 2, pH 7.5 (1.0 M pH 7.5 sodium HEPES;MOPS (acid)), 50% v/v Precipitant Mix 4 (25% v/v MPD; 25% PEG 1000; 25% w/v PEG 3350)	0.06 M Divalents mix (0.3 M magnesium chloride hexahydrate; 0.3 M calcium chloride dehydrate), 0.1 M Buffer System 2, pH 7.5 (1.0 M pH 7.5 sodium HEPES; MOPS (acid)), 50% v/v Precipitant Mix 3 (40% v/v glycerol; 20% w/v PEG 4000)	0.2 M lithium sulfate, 0.1 M Bis-Tris pH 5.5, 25% w/v PEG 3350	0.2 M sodium tartrate dibasic dehydrate, 20 % w/v PEG 3350

Table S1: Table of crystallographic data. PDB codes for the relevant crystal structures are provided. Conditions used to obtain each crystal structure are shown in the final panel.

BM3 Variant	Rhombic LS signals ($g_z/g_y/g_x$)	
	Major species	Minor species
WT	2.41/2.25/1.92	-
WT + DMSO	2.41/2.25/1.91	2.39/2.25/1.93; 2.44/2.25/1.90
WT + Bifonazole	2.47/2.25/1.89	2.57/2.25/1.85
WT + Butoconazole nitrate	2.45/2.26/1.90	2.55/2.26/1.86
WT + Climbazole	2.47/2.26/1.91	2.50/2.26/1.88
WT + Clotrimazole	2.41/2.25/1.91	2.39/2.25/1.93; 2.45/2.25/1.89
WT + Econazole nitrate	2.42/2.26/1.91	2.44/2.26/1.90; 2.52/2.26/1.87; 2.59/2.26/1.84
WT + Fenticonazole nitrate	2.45/2.25/1.91	2.39/2.25/1.94; 2.41/2.25/1.93; 2.53/2.25/2.53
WT + Fluconazole	2.41/2.25/1.91	2.39/2.25/1.93; 2.45/2.25/1.87
WT + Isoconazole nitrate	2.41/2.25/1.91	2.39/2.25/1.92; 2.44/2.25/1.90; 2.52/2.25/1.87
WT + Itraconazole	2.41/2.25/1.91	2.39/2.25/1.92; 2.44/2.25/1.90
WT + Ketoconazole	2.42/2.25/1.91	2.44/2.25/1.90; 2.56/2.25/1.86
WT + Miconazole	2.44/2.25/1.91	2.42/2.25/1.92; 2.52/2.25/1.89
WT + Posaconazole	2.41/2.25/1.90	2.39/2.25/1.93; 2.44/2.25/1.91
WT + Ravuconazole	2.41/2.25/1.90	2.39/2.25/1.93; 2.44/2.25/1.91
WT + Sertaconazole nitrate	2.42/2.25/1.91	2.39/2.25/1.92; 2.44/2.25/1.90
WT + Sulconazole nitrate	2.45/2.25/1.91	2.39/2.25/1.92; 2.56/2.25/1.86
WT + Tioconazole	2.41/2.25/1.91	2.39/2.25/1.93; 2.44/2.25/1.90; 2.53/2.25/1.87; 2.59/2.25/1.85
WT + Voriconazole	2.41/2.25/1.91	2.39/2.25/1.93; 2.44/2.25/1.90

Table S2: **X-band EPR data sets for the WT BM3 heme domain**. Major and minor sets of X-band EPR g-values are shown. Imidazole-containing azoles are shown in green. Triazole-containing azoles are shown in blue.

BM3 Variant	Rhombic LS signals ($g_z/g_y/g_x$)	
	Major species	Minor species
DM	2.44/2.25/1.91	2.41/2.25/1.89
DM + DMSO	2.45/2.25/1.91	2.50/2.25/1.89
DM + Bifonazole	2.49/2.26/1.88	-
DM + Butoconazole nitrate	2.44/2.26/1.89	2.50/2.26/1.88
DM + Climbazole	2.46/2.26/1.89	-
DM + Clotrimazole*	2.46/2.26/1.90	-
DM + Econazole nitrate	2.45/2.26/1.89	2.49/2.26/1.88
DM + Fenticonazole nitrate	2.45/2.26/1.89	2.49/2.26/1.88
DM + Fluconazole*	2.42/2.25/1.91	2.49/2.25/1.89
DM + Isoconazole nitrate	2.46/2.25/1.89	2.44/2.26/1.90; 2.53/2.26/1.88
DM + Itraconazole	2.45/2.25/1.91	2.51/2.26/1.89
DM + Ketoconazole	2.43/2.26/1.90	2.53/2.26/1.87
DM + Miconazole	2.45/2.26/1.90	2.50/2.26/1.87
DM + Posaconazole	2.42/2.26/1.91	2.50/2.26/1.89
DM + Ravuconazole	2.46/2.26/1.89	2.52/2.26/1.87
DM + Sertaconazole nitrate	2.45/2.26/1.90	2.56/2.26/1.88
DM + Sulconazole nitrate	2.45/2.26/1.90	2.56/2.26/1.88
DM + Tioconazole*	2.45/2.26/1.91	2.51/2.26/1.89
DM + Voriconazole*	2.45/2.26/1.90	2.42/2.26/1.91; 2.51/2.26/1.89

Table S3: **X-band EPR data sets for the DM BM3 heme domain**. Major and minor sets of X-band EPR g-values are shown. Imidazole-containing azoles are shown in green. Triazole-containing azoles are shown in blue. The azole drugs indicated by asterisks are those for which crystal structures were obtained.
